# Quality of endoscopic surveillance of Lynch syndrome patients in a Swedish cohort

**DOI:** 10.1055/a-2339-7152

**Published:** 2024-07-04

**Authors:** Sophie Walton Bernstedt, Adrianna Haxhijaj, Nigin Jamizadeh, Jan Björk, Anna Andreasson, Anna M Forsberg, Ann-Sofie Backman

**Affiliations:** 1 27106 Department of Medicine Huddinge, Karolinska Institute, Stockholm, Sweden; 2 59562 Division of Gastroenterology, Dermatology and Rheumatology, Karolinska University Hospital, Stockholm, Sweden; 3 59562 Department of Upper GI Diseases, Karolinska University Hospital, Stockholm, Sweden; 4 27106 Department of Medicine Solna, Karolinska Institute, Stockholm, Sweden; 5 59562 Hereditary Cancer, Medical Unit Breast Endocrine and Sarcoma Tumours, Karolinska University Hospital, Stockholm, Sweden; 6 571139 Department of Medicine Solna, Karolinska Institute Clinical Epidemiology Division, Stockholm, Sweden; 7 83223 Gastroenterology Unit, Division of Medicine, Ersta Hospital, Stockholm, Sweden

**Keywords:** Endoscopy Lower GI Tract, Polyps / adenomas / ..., Colorectal cancer, Quality and logistical aspects

## Abstract

**Background and study aims** Risk factors for colorectal cancer (CRC) in Lynch syndrome (LS) include sex, age, smoking, high body mass index (BMI), surveillance interval length, and risk genotype. The Boston Bowel Preparation Scale (BBPS) produces a standardized bowel cleanliness rating. A low BBPS score might be a risk factor for missed early lesions. The aim of this study was to investigate the correlation between BBPS score and adenoma detection (with known risk factors for CRC) and surveillance interval with CRC detection in LS patients.

**Methods** A retrospective cohort study including 366 LS patients with 1,887 colonoscopies under surveillance in Stockholm, Sweden from 1989 to 2021 was conducted. Associations were tested using linear and logistic regression.

**Results** We found no association between BBPS score and number of adenomas detected. A low BBPS score was found to be associated with older age (regression coefficient (coeff) –0.015; 95% confidence interval [CI] –0.026 to –0.004; *P* = 0.007) and obesity (coeff = –0.48; 95% CI: –0.89 to –0.062; *P* = 0.024). A higher number of detected adenomas was associated with older age (coeff = 0.008; 95% CI 0.004 to 0.012; *P* < 0.001), male sex (coeff = 0.097; 95% CI 0.008 to 0.19; *P* = 0.033) and CRC (coeff = 0.28; 95% CI 0.061 to 0.50; *P* = 0.012). Surveillance interval length was not significant in CRC detection.

**Conclusions** Bowel cleanliness was not associated with adenoma detection and was less likely achieved in patients who were older and had higher BMI. Adenoma detection was associated with older age and male sex. The results indicate the need for better adherence to guidelines and attention to older age groups, men, and patients with obesity.

## Introduction

Lynch syndrome (LS) is caused by a mismatch repair (MMR) deficiency and it is a genetic condition that greatly increases risk of colorectal cancer (CRC). Before a genetic cause was identified, patients were identified as having hereditary nonpolyposis CRC (HNPCC) by clinical criteria such as the Amsterdam or Bethesda criteria 1 2 . Regular colonoscopies are recommended to detect early-stage CRC and remove premalignant lesions 3 , with the recommended interval being 2 years according to the European Society of Gastrointestinal Endoscopy in 2019 (ESGE) 4 .

The high lifetime CRC risk in LS is primarily dependent on the genotype. Carriers of pathogenic variants in the MLH1 and MSH2 genes are estimated to have a similarly high risk of developing CRC, MSH6 carriers have an intermediate risk on this genetic spectrum 5 , and PMS2 carriers a lower risk 6 . Cigarette smoking 7 and a high body mass index (BMI) 8 were also linked to an increased risk of CRC in the general population, whereas male sex, smoking, and a high BMI were linked to an increased risk of CRC among LS patients in a Swedish cohort 9 . In this study, MLH1 and MSH2 carriers were shown to maintain the above-mentioned elevated risk of CRC, despite endoscopic surveillance 9 . Considering this, it is essential to investigate if known risk factors for CRC also are associated with bowel cleanliness, and in turn, might influence adenoma detection, and thereby, an elevated risk of missed early cancer.

To this day, the factors that affect CRC risk in LS, as well as the extent of their effects, have not been fully explored. In addition, the extent to which individual versus organizational factors are associated with colonoscopy outcomes is also poorly understood. Thus, it is crucial to identify these factors to enable optimal and personalized endoscopic surveillance.

Optimal visualization of bowel mucosa is essential for adenoma detection, and its success is dependent on a clean colon. According to the ESGE 10 , the Boston Bowel Preparation Scale (BBPS) is the most reliable and clinically relevant method for rating bowel cleanliness 11 and with it, each colon segment – the right, transverse, and left colon – is given a score of 0 to 3, for a maximum possible total score of 9 points, with higher scores reflecting better preparation. Further, a higher BBPS score is correlated with a higher adenoma detection rate (ADR), defined as the percentage of all colonoscopies in which at least one colorectal adenoma was detected 12 . CRC detected during surveillance in LS patients has previously been shown not to be preceded by a colonoscopy of poor quality 13 .

The primary aim of this study was to investigate the correlation between BBPS score and adenoma detection. Secondary aims were to investigate correlations between BBPS score, adenoma detection, and known risk factors for CRC and to calculate the correlation between surveillance interval and CRC detection in LS patients.

## Patients and methods

### Study design and subjects

A single-center observational cohort study was conducted in Stockholm, Sweden, at Karolinska University Hospital, whose catchment area primarily includes Stockholm County, although the hospital also acts as a second-opinion facility for northern and middle Sweden. Further, approximately one-quarter of Swedish LS patients are diagnosed at Karolinska University Hospital 14 , with follow-up care available at several endoscopic centers in Stockholm (the majority at Karolinska University Hospital).

In 2018, the population of Stockholm County was 2.3 million 15 , almost one-quarter of the total population of 10 million in Sweden 16 . The study included LS patients with an MMR gene mutation that is registered at the Karolinska University Hospital and that was confirmed according to the InSiGHT Variant Interpretation Committee classification 17 or reported by the hospital’s genetics department if the variant at the time was unknown. This cohort has been described previously 9 18 .

### Data collection

LS patients undergoing endoscopic surveillance in Stockholm County from August 1989 to April 2021 were included in the study and related medical data (such as cancer diagnosis) were available from 1975 to 2021. Data on patient characteristics (age, sex, genotype, smoking habits, and BMI) were collected using standardized protocols at the index medical visit.

Endoscopic data regarding BBPS score and number of adenomas at each colonoscopy were collected in a separate standardized protocol. Only complete and documented BBPS scores, determined according to endoscopist evaluation, were included. Mean number of adenomas at each colonoscopy (MAC) was calculated as the total number of adenomas detected in a patient across all colonoscopies divided by the number of colonoscopies the patient had completed. Endoscopic interval was defined as time between two surveillance colonoscopies. Interval cancer was defined as CRC diagnosed ≥ 3 months after negative colonoscopy (clean colon) but before the next planned surveillance. Index colonoscopy was defined as the first surveillance colonoscopy after a patient had received a genetic diagnosis or a clinical diagnosis of HNPCC. Only surveillance colonoscopies on the entire colorectum were included in the analyses. At Karolinska University Hospital, various endoscopists performed the procedures over the study period, and there are no data about specific endoscopist ADR or the total number of endoscopists performing the procedures.

Smoking status was defined as previous/current smoker or non-smoker and obesity was defined as BMI ≥ 30.

### Statistics

To test the association between interval and CRC detection, mixed effect logistic regression analysis clustered on patient identity was used.

Mixed effects multivariable linear regression with BBPS score as dependent variable and age, sex, smoking status, and overweight status as independent variables clustered on patient identity as a random effect was used to test factors associated with bowel cleanliness at colonoscopy. In this analysis, all available protocols were used and patient identity was used as random effect to account for the fact that some patients contributed with several procedures. This analysis included 345 protocols across 200 patients. To test the association between BBPS and adenoma detection, mixed effects linear regression was used in a similar fashion.

To test the association between patient factors and MAC, multivariable linear regression was used with MAC as a dependent variable and age, genotype, sex, smoking status, obesity status, and CRC detected during surveillance as independent variables. This analysis included 267 patients with complete data; a total of 99 patients were excluded (colorectal surgery or no documented colonoscopies). Age, smoking status, and obesity status from the index medical visit was used.

All analyses were bootstrapped with 2000 repetitions to account for non-normal distribution of BBPS score and MAC. Statistical significance was set at P < 0.05 and all statistical calculations were produced in STATA (version 18) for PC.

### Ethics

All procedures performed were part of routine clinical care. The Regional Ethics Review Board of Stockholm approved this study (Dnr 2017/2013–31/2 and Dnr 2022–00119–02).

## Results

In total, 366 patients with confirmed pathogenic MMR gene variants were included in the study and the characteristics of the study population are described in Table 1 .

**Table:** **Table 1** Study population characteristics.

**Study population, n**	**366**
**Deceased, n (%)**	15 (4)
**Age at genetic diagnosis, mean years (range)**	42 (16–93)
**Genotype**	
*MLH1* , n (%)	164 (45)
*MSH2* , n (%)	103 (28)
*MSH6* , n (%)	51 (14)
*PMS2* , n (%)	38 (10)
*EPCAM* , n (%)	6 (2)
Mixed genotype, n (%)	4 (1)
**Gender**	
Women, n (%)	197 (54)
Men, n (%)	169 (46)
**Smoking**	
Current smoker, n (%)	36 (10)
Previous smoker, n (%)	104 (28)
Never-smoker, n (%)	210 (57)
Missing data, n (%)	16 (4)
**BMI, mean (range)**	25.4 (16.6–49.5)
**Chemoprevention**	
Current or previous, n (%)	47 (13)
Never, n (%)	295 (81)
Missing data, n (%)	24 (7)
**CRC diagnosis n (%)**	108 (30)
**Age at CRC diagnosis, mean years (range)**	45 (22–79)
BMI, body mass index; CRC, colorectal cancer.	

### Surveillance

In total, 1,887 endoscopies were recorded, of which 1,334 were performed on the entire colorectum ( Table 2 ). Of these procedures, 348 endoscopies were performed as index procedures. Complete BBPS score was documented in 345 colonoscopies (26%), for which the mean BBPS score was 7.7. Of colonoscopies with a complete BBPS score, 63% were performed with a preparation BBPS score of 8 to 9, 33% a score 6 to 7 and 4% a score < 6.

The mean surveillance interval was 19 months and that for CRC detection was 24 months. Interval was not significant in CRC detection in this cohort (odds ratio 1.02; 95% confidence interval [CI] 0.99–1.04; *P* = 0.17).

**Table:** **Table 2** Characteristics of surveillance colonoscopies in Lynch syndrome (LS) patients.

**Endoscopies 1989–2021, n**	**1,887**
**Colonoscopies (the entire colorectum), n**	1,334
**Interval ≤ 24 months, n**	1,154
**Interval > 24 months, n**	365
**Index endoscopies, n (%)**	348 (95%)
**Age at index colonoscopy, mean years (range)**	40 (18–82)
**BBPS score, mean (range)**	7.7 (3–9)
**Colonoscopies documented with complete BBPS score, n (%)**	345 (26%)
**BBPS score < 6 (n, %)**	13 (4%)
**BBPS score 6–7 (n, %)**	115 (33%)
**BBPS score 8–9 (n, %)**	217 (63%)
**Number of adenomas, individual investigations, range**	0–8
**MAC, mean (range)**	0.2 (0–2)
**Colonoscopies with documented BBPS score and adenoma detection, n (%)**	55 (15.9%)
**Colonoscopies without documented BBPS score and adenoma detection, n (%)**	155 (15.7%)
**Endoscopic interval (months), mean (range)**	19 (3–150)
**Within 18 months**	57%
**Within 24 months**	76%
BBPS, Boston Bowel Preparation Scale.	

### Cancer detection

CRC was detected in 10 of 348 index colonoscopies ( Table 3 ), and among the 1,539 colonoscopies performed after the index procedures, 18 cases of CRC were found, of which seven (37%) were in patients with a surveillance interval between clean colonoscopies longer than 24 months. Of the 18 CRCs detected during surveillance, 14 were in *MLH1* carriers and four in *MSH2* carriers. Only one colonoscopy had a documented structured BBPS score preceding CRC detection.

**Table:** **Table 3** Detection of colorectal cancer and mean number of adenomas per colonoscopy during endoscopic surveillance in Lynch syndrome.

**CRC detected at surveillance, n**	**28**
**CRC detected at index, n**	10
**CRC detected during surveillance (interval ≤ 24 months), n**	11
**CRC detected during surveillance (interval > 24 months), n**	7
**Endoscopic interval to detection of cancer, mean months (range)**	24 (3–71)
**MAC before CRC diagnosis, n (range)**	0.5 (0–1.7)
CRC, colorectal cancer; MAC, mean number of adenomas at each colonoscopy.	

### Endoscopic quality

BBPS scores were recorded for procedures performed from 2015 to 2021, where an association between low BBPS score and older age was observed (regression coefficient –0.015; 95% CI –0.026 to –0.004; P = 0.007) and obesity (coefficient –0.48; 95% CI –0.89 to –0.062; P = 0.024) ( Table 4 ). There was no statistically significant difference in number of detected adenomas and BBPS score (coefficient –0.023; 95% CI –0.064 to 0.017; P = 0.26).

**Table:** **Table 4** Boston Bowel Preparation Scale and patient-related factors.

**Risk factor**	**Observed coefficient**	**95% CI**	***P***	**Ref (n)**
Age (linear)	–0.015	(–0.026 to –0.004)	0.007	–
Sex	0.091	(–0.15 to 0.33)	0.46	Men (89/200)
Smoking*	–0.23	(–0.52to 0.061)	0.12	Non-smokers (125/195)
BMI†	–0.48	(–0.89 to –0.062)	0.024	Non-obesity (176/198)
Mixed effects multivariable linear regression with BBPS as outcome variable and age, sex, smoking, and obesity clustered on patient identity was used. Two hundred patients with 345 colonoscopies were included in the analysis. *Smoking association (current and previous smokers) was compared with non-smokers †Obesity (BMI ≥ 30) was compared with non-obesity (BMI < 30). BBPS, Boston Bowel Preparation Scale; BMI, body mass index.				

### Adenoma detection and MAC

The number of adenomas detected varied between 0 and 8 (range) in the individual investigation ( Table 3 ) and the overall MAC in the cohort was 0.2 (range 0–2). The ADR for investigations in which a complete BBPS score was noted was 15.9%, whereas the ADR for colonoscopies that lacked a complete BBPS score was 15.7%. Older age (coefficient 0.008; 95% CI 0.004 to 0.012; P < 0.001), male sex (coefficient 0.097; 95% CI 0.008 to 0.19; P = 0.033), and CRC detected during surveillance (coefficient 0.28; 95% CI 0.061 to 0.5; P = 0.012) were associated with a higher MAC ( Table 5 ), which was 0.18 for *MLH1* carriers, 0.23 for *MSH2* carriers, 0.23 for *MSH6* carriers, 0.19 for *PMS2* carriers, 0.13 for *EPCAM* carriers, and 0.40 for carriers of a mixed genotype (range 0–2).

**Table:** **Table 5** Associations between patient-related risk factors and mean adenomas per colonoscopy.

**Mean adenomas/colonoscopy**	**Observed coefficient**	**95% CI**	***P* value **	**Ref (n)**
**Age (linear)**	0.008	(0.004–0.012)	< 0.001	–
**Genotype**				MLH1 (120/267)
	0.01	(–0.087 to 0.11)	0.85	MSH2 (73/267)
	0.022	(–0.12 to 0.16)	0.76	MSH6 (36/267)
	–0.018	(–0.17 to 0.13)	0.82	PMS2 (30/267)
	0.039	(–0.098 to 0.18)	0.58	EPCAM (5/267)
	0.2	(–0.20 to 0.60)	0.33	Mixed (3/267)
**Sex**	0.097	(0.008 to 0.19)	0.033	Men (119/267)
Smoking*	0.094	(–0.006 to 0.19)	0.064	Non-smokers (165/254)
**BMI†**	–0.093	(–0.25 to 0.065)	0.25	Non-obesity (230/259)
**Colorectal cancer during surveillance**	0.28	(0.061 to 0.5)	0.012	Yes (22/267)
Multivariable linear regression analysis with MAC as outcome variable and age, genotype category, sex, smoking, and obesity at index medical visit was used. Data from 267 patients were included in the analysis. *Smoking association (current and previous smokers) was compared with non-smokers †Obesity (BMI ≥ 30) was compared with non-obesity (BMI < 30). CI, confidence interval; MAC, mean adenomas at each colonoscopy; BMI, body mass index.				

## Discussion

The primary aim of this study was to investigate the correlation between BBPS score and adenoma detection; secondary aims were to investigate correlations between BBPS score, adenoma detection, and known risk factors for CRC, and to calculate the correlation between surveillance interval and CRC detection in LS patients in Stockholm from 1989 to 2021.

In this cohort, the BBPS score was recorded correctly in only 26% of colonoscopies, because this classification was introduced as a standard late in the study period. Our findings show that a low BBPS score was associated with advanced age and high BMI, but not with sex or smoking, and we did not find any association between BBPS score and number of adenomas. Older age, male sex, and CRC detected during surveillance were associated with increased MAC.

Interval cancer was only observed in *MLH1* and *MSH2* carriers in our study population, suggesting that these genotypes require a shorter surveillance interval if the purpose of surveillance is to prevent CRC; currently, the ESGE recommends a 2-year interval for all genotypes 4 . In this cohort, interval length was not significant to CRC detection. Adenoma detection did not vary with BBPS score, suggesting that other endoscopic factors, such as technique and methods, may play a more important role. However, the lack of significance may be due to the small number of reported BBPS scores. The BBPS score was previously documented in an unstandardized form 19 , reflecting that the endoscopic data in this study were collected from 1989 onward, but use of BBPS score was only introduced recently. A high BBPS score has been shown to correlate with younger age 20 , consistent with this study. The same study 20 also found that the strongest predictors for identifying pathologic findings were age and male sex, and a older age and male sex in this study were associated with higher MAC. As many as one-third 21 of patients undergoing colonoscopy may have an inadequately prepared bowel, the risk factors for which in one study 21 were older age, BMI > 25, low fruit consumption, and history of smoking. Use of nonsteroidal anti-inflammatory drugs and selective serotonin reuptake inhibitors was correlated with higher bowel preparation scores 21 , and when used, BBPS score was also subject to individual endoscopist interpretation. Other endoscopic factors, such as colonoscopy withdrawal time 22 and use of chromoendoscopy 23 , may be more important for adenoma detection than BBPS score. Moreover, surveillance intervals less than 3 years in the same study were associated with a reduction in post-colonoscopy CRC incidence 23 . Women have previously been shown to have better BBPS scores than men, and cecal intubation times were longer in women. Withdrawal time might be important for polyp detection, although other factors, such as sex, may impact adenoma detection and thereby influence ADR 24 , which has previously been shown to be higher in men than women 25 26 . Whether the observed MAC is due to the genetic risk itself or to the fact that adenomas are sporadic and related to age cannot be determined using our data.

Obesity in LS has shown an association with increased CRC risk and an overall increased risk of LS-associated cancers, and it seemed to increase CRC risk, particularly in patients with pathogenic *MLH1* variants 27 . Studies have shown that men with LS have an increased CRC risk compared with women 9 28 . LS patients may increase their risk of CRC if they smoke regularly, and former smokers potentially have a lower CRC risk compared with never-smokers 29 . However, it has also been demonstrated that CRC risk may be increased among former smokers 9 .

### Strengths

The major strength of this study is that it was a large cohort, considering the rarity of the genetic condition. Data were compiled about characteristics of 366 patients and correlated with surveillance data from their consecutive 1,887 endoscopies over 31 years of follow-up. Most patients underwent all their endoscopic procedures at the same hospital in the Gastroenterology Outpatient Clinic (Karolinska University Hospital), which made data collection by protocols more uniform.

### Limitations

The limitations include the lack of data collected about which type of laxative the patients were prescribed and how well the preparations worked for the patients. Because both BBPS score and adenoma detection are subjective variables dependent on the endoscopist, inter-investigator variability may be present. However, the data are considered more dependable than a retrospective classification performed on pictures from procedures, because pictures are most often taken of the part of the gut where visibility is best, which may result in an inaccurate representation. Further, the lack of significant associations may be due to the few procedures that used BBPS score, documented in only 345 of 1,334 complete surveillance colonoscopies because the score was introduced in 2009 only and had not been fully implemented at the start of the study period 11 . We chose not to include the older system, where subjective descriptions such as “adequate or inadequate bowel preparation” were used because of the risk of introducing bias. Introduction of high-definition colonoscopy in recent years has also increased adenoma detection 30 .

In addition, data on smoking and BMI were measured at one time point only, and there is a limitation in that the level of exposure to such risk factors as smoking was not designated as a specific amount. There is also a potential for recall bias in obtaining information about risk factors (such as smoking) from patients who have had cancer.

## Conclusions

In this group of LS patients with an MMR gene mutation, the CRC detection rate was higher after 24 months, although it was not statistically significant. Achieving adequate bowel cleanliness was less likely in individuals who where older and and had higher BMI, and a higher MAC was associated with older age, male sex, and CRC detected during surveillance. BBPS was not associated with the number of adenomas detected during endoscopic surveillance. Still, individual and procedure risk factors seem to play a role in adenoma detection, which may support individualized surveillance intervals. These results indicate the need for better adherence to guidelines regarding surveillance intervals and special attention to older age groups, men, and obese individuals in regard to preparation.

### Data availability statement

The raw data supporting the conclusions from this article will not be made available by the authors due to patient confidentiality.
